# Exploring the differences between the three pyruvate kinase isozymes from *Vibrio cholerae* in a heterologous expression system

**DOI:** 10.1186/s13104-018-3651-8

**Published:** 2018-07-31

**Authors:** Zoe Alba-Martínez, Leticia Ramírez-Silva, Gloria Hernández-Alcántara

**Affiliations:** Departamento de Bioquímica, Facultad de Medicina, Universidad Nacional Autónoma de México, 04510 Mexico City, Mexico

**Keywords:** *E. coli* toxic gene, Transformation, Plasmid stability, Protein expression, *Vibrio cholerae*, Pyruvate kinase genes

## Abstract

**Objective:**

The genome of *Vibrio cholerae* has three paralog genes encoding for distinct pyruvate kinases. We were interested in elucidating whether they were expressed, and contributed to the pyruvate kinase activity of *V. cholerae. Vc*IPK and *Vc*IIPK were transformed and expressed in BL21-CodonPlus(DE3)-RIL strain, whereas *Vc*IIIPK could not be transformed. Those studied did contribute to the pyruvate kinase activity of the bacteria. Therefore, our aim was to find an efficient transformation and commonly used over-expression heterologous system for *Vc*IIIPK and develop its purification protocol.

**Results:**

*vcIpk*, *vcIIpk* and *vcIIIpk* genes were transformed in six different BL21 expression strains. No transformants were obtained for the *vcIIIpk* gene using BL21(DE3), BL21(DE3)pLysS and BL21(DE3)CodonPlus-RIL strains. Reduced rates of cell growth were observed for BL21-Gold(DE3)pLysS and Origami B(DE3)pLysS. High efficiency of transformation was obtained for BL21-AI. Using this strain, *Vc*IIIPK was purified but proved to be unstable during its purification and storage. Therefore, the transformation of *vcIIIpk* gene resulted in a toxic, mildly toxic or nontoxic product for these BL21 strains. Despite *Vc*IIPK and *Vc*IIIPK being phylogenetically related, the preservation of the proteins is drastically different; whereas one is preserved during purification and storage, the other is auto-proteolyzed completely in less than a week.

**Electronic supplementary material:**

The online version of this article (10.1186/s13104-018-3651-8) contains supplementary material, which is available to authorized users.

## Introduction

*Vibrio cholera*e is a gram-negative bacterium that causes the acute secretory diarrheal disease named cholera. This pathogen exhibits an extraordinary ability to rapidly evolve in a changing environment [1]. Genome plasticity and horizontal gene transfer allows *V. cholerae* to survive in a multitude of different environments [2]. Its genome contains two circular chromosomes encoding nearly 4000 open reading frames distributed between the large (2.96 Mb) and the small (1.07 Mb) chromosomes [3, 4]. *V. cholerae* has three open reading frames encoding for distinct pyruvate kinases (PKs); *Vc*IPK and *Vc*IIPK are present in the large, whereas *Vc*IIIPK is in the small chromosomes, respectively. Sequence alignments show that the amino acid identity between *Vc*IPK and *Vc*IIPK is 37%, between *Vc*IPK and *Vc*IIIPK is 36% whereas between *Vc*IIPK and *Vc*IIIPK is 50% (Additional file 1: Table S1). However, up to date there are no reports if the *Vc*PKs are expressed differentially in distinct environmental conditions or what metabolic role does each PK have in *V. cholerae*.

In a recent study, *Vc*IPK and *Vc*IIPK were transformed and expressed in BL21-CodonPlus(DE3)-RIL. Purified *Vc*IPK and *Vc*IIPK were kinetically characterized. It was also identified by Western blot analyses that both enzymes are present in cell extracts of CVD103 *V. cholerae* strain. Since they co-express, and their catalytic requirements are present in the bacterium, it was concluded that both enzymes contribute to the activity of pyruvate kinase in *V. cholerae* [5]. However, *vcIIIpk* gene failed to transform in BL21-CodonPlus(DE3)-RIL. Thus, *Vc*IIIPK was not included in that study. Therefore, the aim of this study was to find a bacterial expression system for the efficient transformation and over-expression of *Vc*IIIPK and to design a purification protocol for the enzyme to elucidate whether *Vc*IIIPK contributed to the activity of PK of *V. cholerae*. For this purpose, we tested 6 strains of the BL21 expression system. The BL21DE3 system is commonly used for over-expression of heterologous genes due to the simple transformation, manipulation, rapid growth of cells and high yield of protein [6, 7]. We found that the *vcIIIpk* gene was toxic, mildly toxic and nontoxic for 3, 2 and 1 strains, respectively.

## Main text

### Methods

Methods used in this study are provided in Additional file 2.

### Results and discussion

The *vcIIIpk* gene (1461 bp) was cloned into plasmid pET3a-HisTEVP. *vcIpk* and *vcIIpk* genes [5] were transformed into several BL21(DE3) strains to compare against the transformation of *vcIIIpk*. DE3 strains have a chromosomal copy of the phage T7 RNA polymerase gene that is compatible with the pET vectors, for which the genes are cloned downstream of the strong T7 promoter being used for recombinant protein expression [6, 8–10]. Six BL21 expression strains: BL21(DE3), BL21(DE3)pLysS, BL21(DE3)CodonPlus-RIL, BL21-Gold(DE3)pLysS, OrigamiB(DE3)pLysS and BL21-AI with different characteristics (Additional file 3: Table S2), were separately transformed with the constructs of the three *vcpk* genes. Five strains contain the DE3 system, whereas BL21-AI strain is controlled by the arabinose operon (araBAD promoter) [11].

#### *vcIIIpk* gene yields no transformants in some BL21 strains

BL21(DE3), BL21(DE3)CodonPlus-RIL and BL21Gold-(DE3)pLysS strains (Additional file 3: Table S2) were transformed with the three constructions of *vcpks*. The results showed that, for *vcIIIpk* gene, no colonies were obtained with these strains, whereas *vcIpk and vcIIpk* genes were positively transformed (Additional file 4: Figure S1). The absence of transformants may be explained due to plasmid instability in these strains or because the over-expression of target gene is toxic for them due to the metabolic burden [12–14]. To probe the instability of the *vcIIIpk* construct into these strains, the three genes were transformed into the non-expression host XL10-GOLD strain, commonly used for the propagation and manipulation of recombinant DNA. As expected, all three genes were efficiently transformed (Additional file 4: Figure S1). Therefore, it was inferred that the gene would probably be toxic for these expression bacteria [15]. BL21(DE3)CodonPlus-RIL strain was used to transform *vcIIIpk* gene, due to the successful transformation of *vcIpk and vcIIpk* genes in this strain [5]. BL21Gold-(DE3)pLysS strain was used due to its property of high efficiency of transformation and BL21(DE3) was used to elucidate if the gene was toxic.

#### *vcIIIpk* gene transformants with reduced rates of cell growth

BL21(DE3) with pLysS or lysY strain is recommended when no transformants are found. pLysS or lysY strains may yield normal colonies and express the protein of interest in moderate to high levels. Samuelson [16] describes that mildly toxic gene products may be lethal for BL21(DE3) upon transformation. When *vcIIIpk* gene was transformed into BL21(DE3)pLysS strain, different colony sizes were observed. Some transformants displayed normal growth, while most transformants grew weakly. Growth rates were also affected; colonies grew in 20 h to over 24 h. In contrast, no such effects were observed when the same strains were transformed with *vcIpk* or *vcIIpk* genes (Additional file 4: Figure S1).

OrigamiB(DE3)pLysS strain is recommended to enhance disulfide bond formation in the cytoplasm. Several studies have shown that the expression in this strain yields tenfold more active proteins than in other hosts [17], even though the level of overall expression is similar. Since *Vc*IIIPK has 8 Cys/monomer with unknown role, the gene was transformed in this strain (Additional file 5: Table S3). The efficiency of transformation of the three genes was low but colonies of *vcIpk* and *vcIIpk* were observed after an overnight culture, whereas *vcIIIpk* transformants delayed for 72 h (Additional file 4: Figure S1). After this time, the shape and size of the colonies were similar to those obtained with the other genes. Reduced rates of cell growth indicated that *vcIIIpk* gene was mildly toxic for these strains.

#### *vcIIIpk* gene transformants with typical rates of cell growth

BL21-AI strain is especially useful to express genes that may be toxic to other strains of group BL21. This strain carries a chromosomal insertion of a cassette containing the T7-RNA polymerase gene in the *ara*B locus, allowing expression of the T7-RNA polymerase to be regulated by the *ara*BAD promoter [18, 19]. The efficiency of transformation of *vcIIIpk* gene in BL21-AI strain increased markedly. After an overnight culture, homogeneous size colonies were observed (Additional file 4: Figure S1). Therefore, in this strain *vcIIIpk* gene is not toxic, contrasting with the strains under control of the T7 promoter.

In sum, these results indicate that the same strategy may not always be the same for expressing paralog genes.

#### Protein expression screening of *Vc*IIIPK in BL21 strains

In order to understand the distinct level of toxicity caused by *vcIIIpk* gene in BL21(DE3)pLysS, OrigamiB(DE3)pLysS and BL21-AI strains, the protein expression screening of *Vc*PKs was achieved.

**Fig. 1:**
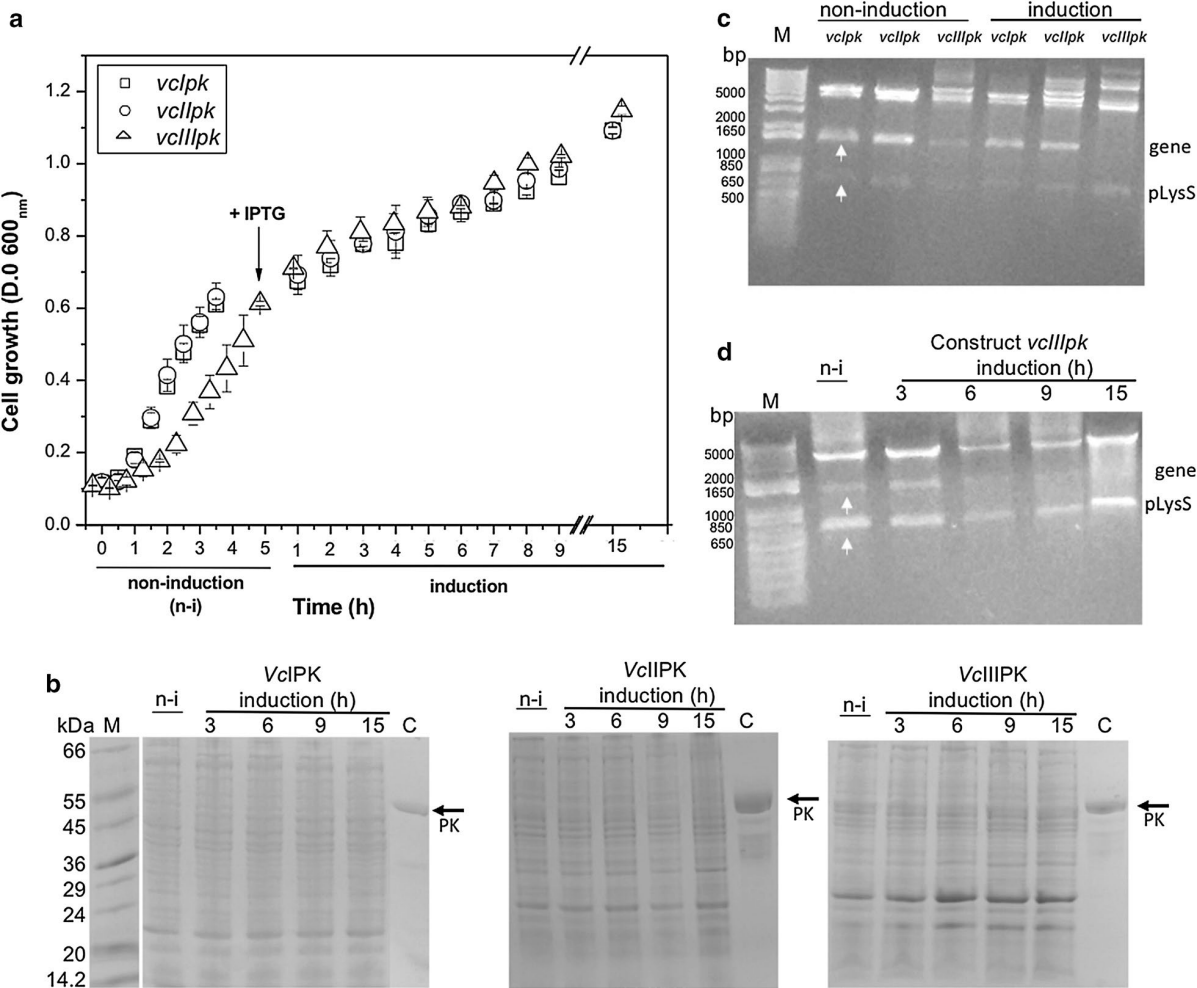
*Vc*IIIPK in BL21(DE3)pLysS strain. **a** Time course of growth curves of *vcIpk*, *vcIIpk* and *vcIIIpk* constructs before (non-induction) and after induction with 0.6 mM IPTG. The error bars represent the standard deviation of three independent experiments. **b** SDS-PAGE gels (12%) show the time course of *Vc*IPK, *Vc*IIPK or *Vc*IIIPK before and after induction. The migration of a previously purified *Vc*IPK used as marker is shown in Lane C and the low range molecular weights (Sigma Marker) are in lane M. **c** Agarose gel (1%) of the *vcIpk*, *vcIIpk* and *vcIIIpk* constructs before and after 15 h of induction. DNAs were digested with NdeI and BamH1. Two bands of ~ 1650 and ~ 650 bp corresponded to the PK genes and to a fragment of pLysS vector, respectively. **d** Agarose gel (1%) of the time course of *vcIIIpk* construct before and 3, 6, 9 and 15 h after induction. DNA was digested as in **c** and the same bands pattern was observed. In **c** and **d** 1 Kb Plus DNA ladder (Invitrogen) were used as markers (M). **b**–**d** Performed from one of the experiments of **a**

The BL21(DE3)pLysS strain carrying the three genes were grown at 37 °C. Cells were induced and monitored hourly. Growth curves were similar for the BL21(DE3)pLysS strain carrying *vcIpk* and *vcIIpk* genes, whereas the growth curve of that carrying *vcIIIpk* gene was delayed for 2 h. After induction, the three growth curves were similar (Fig. 1a). To monitor the protein expression at different times of induction, aliquots of cells were loaded onto SDS-PAGE. As shown in Fig. 1b, no bands, with the expected molecular mass (Additional file 5: Table S3), were observed for the three isozymes.

In order to confirm the stability of the plasmid in BL21DE3pLysS strain during the growth curve, restriction enzyme analyses were performed (Fig. 1c). The restriction map before induction showed two bands, one corresponded to the respective construct (~ 1650 bp) and the other corresponded to the product of the two restriction sites for BamHI of the plasmid pLysS (~ 650 bp (Fig. 1c). 15 h after induction, the two bands of *vcIpk and vcIIpk* constructs were still observed, whereas in the *vcIIIpk* construct, only the fragment of the plasmid was observed (Fig. 1c). Concerning this result, the presence of *vcIIIpk* gene was monitored at different times after the addition of IPTG. As shown in Fig. 1d, *vcIIIpk* gene was present after 3 h of induction, but completely disappeared between 6 and 15 h after induction.

**Fig. 2:**
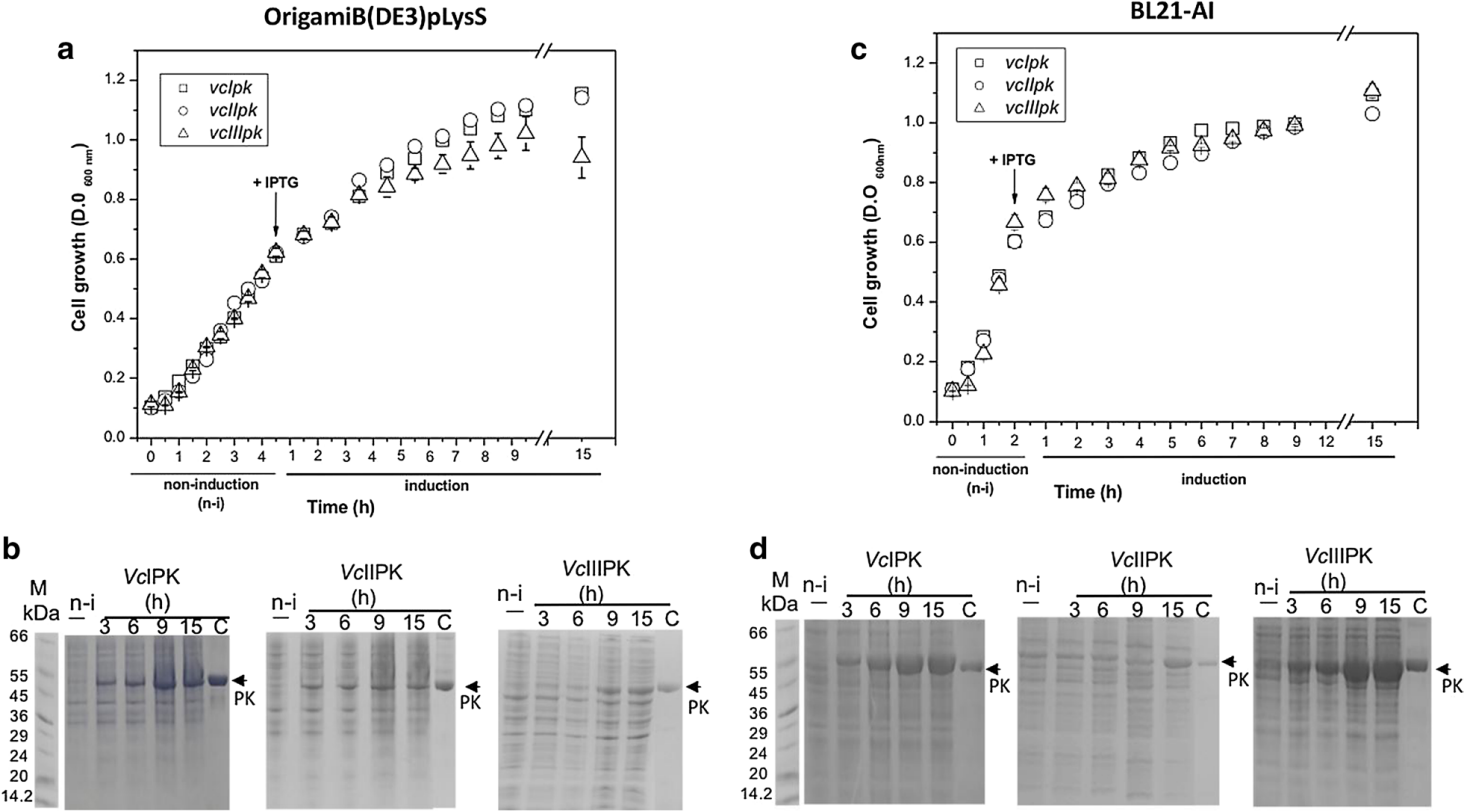
*Vc*IPK, *Vc*IIPK and *Vc*IIIPK in Origami B(DE3)pLysS (**a**, **b**) and in BL21-AI (**c**, **d**) strains. Time course of growth curves of *vcIpk*, *vcIIpk* and *vcIIIpk* constructs before (non-induction) and after induction with 0.6 mM IPTG at 37 °C (**a**) or 1.2 mM IPTG plus 0.25% L-arabinose at 25 °C (**c**). The error bars represent the standard deviation of three independent experiments. **b** and **d** SDS-PAGE (12%) show the time course of *Vc*IPK, *Vc*IIPK or *Vc*IIIPK before (n-i) and after 3, 6, 9 or 15 h of induction. The migration of a previously purified *Vc*IPK used as marker is shown in Lane C and the low range molecular weights (Sigma Marker) are in lane M

The growth curves of OrigamiB(DE3)pLysS strain carrying the three genes were similar before and after induction with IPTG (Fig. 2a). As shown in Fig. 2b all proteins were expressed. It has been shown that expression of proteins in Origami strains yield ten-fold more active proteins than in other strains [17, 20].

The growth curves of the three PKs in BL21-AI strain were similar before and after induction (Fig. 2c). The cultures of the three PKs reached an OD_600=_0.6 in 2 h, whereas in OrigamiB(DE3)pLysS it took 5 h. In BL21-AI strain, *Vc*IPK and *Vc*IIIPK were expressed more intensely than *Vc*IIPK (Fig. 2d) and also better than in OrigamiB(DE3)pLysS (Fig. 2b). Therefore, *Vc*IIIPK was not toxic for these strains. BL21-AI strain contains an arabinose promoter which exhibits the lowest basal transcriptional activity [18, 21, 22]. This feature is important for the maintenance of any toxic gene. This strain is suitable for high-level expression of a recombinant protein from any T7-based expression vector. Because T7 RNA polymerase levels can be tightly regulated, this strain is recommended to express genes that may be toxic to other BL21 strains [15].

#### Protein expression and purification in BL21-AI strain

**Fig. 3:**
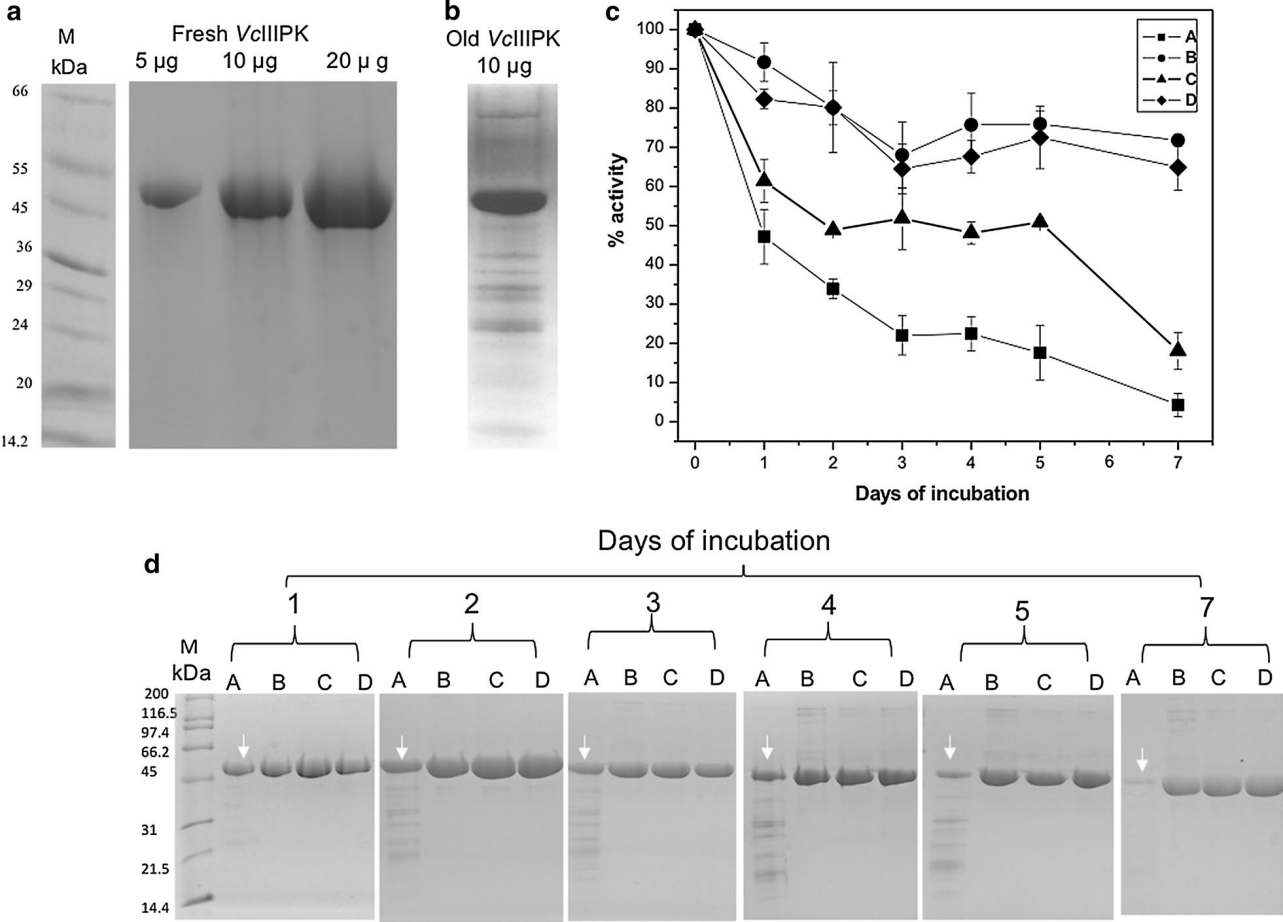
Purification of *Vc*IIIPK in BL21-AI strain (**a**, **b**) and preservation of the purified enzyme (**c**, **d**). **a** SDS-PAGE (12%) of a freshly purified *Vc*IIIPK at different protein concentrations and **b** the same desalted sample of *Vc*IIIPK after a month (old *Vc*IIIPK) of being stored with 80% ammonium sulfate. Lane M indicates the low range molecular weights (Sigma Marker). **c** Residual activity and **d** SDS-PAGE (12%) of *Vc*IIIPK after the first days of purification and storage in different conditions: A. without glycerol at 4 °C, B. 20% glycerol at 4 °C, C. 20% glycerol at − 20 °C and D. 20% glycerol at − 70 °C. 100% of activities without and with glycerol were 176 ± 29 and 185 ± 23 μmoles/min mg, respectively. In **c** the error bars represent the standard deviation of three independent experiments. **d** Performed from one of the experiments of **c**. Lane M shows the broad range molecular weights (BIO-RAD)

*Vc*IIIPK was purified from a BL21-AI strain culture as described in [5]. After the purification, an SDS-PAGE showed a single band of approximately 50 kDa (Fig. 3a). This protein was precipitated with 80% of ammonium sulfate. After a month, the protein was desalted and showed a new pattern of bands in an SDS-PAGE (Fig. 3b). Since the protocol of purification was carried out with a complete protease inhibitor cocktail and 0.2 mM PMSF to prevent proteolysis, it was suspected that *Vc*IIIPK could be auto-proteolyzed during the purification. BL21-AI strain is an *E. coli* B/r protease deficient strain (Additional file 3: Table S2). To prevent the protein from being proteolyzed, glycerol was added during (10% v/v) and after the purification (20% v/v). In the absence of glycerol, *Vc*IIIPK lost activity during the purification and storage, and became inactive after a week of being stored and the band almost completely disappeared (Fig. 3c, d). To achieve the best storage conditions, the protein with 20% of glycerol was kept at − 20, − 70 and 4 °C. After a week stored at 4 °C, the protein remained with 70% of its activity (Fig. 3c) and higher molecular weight bands were observed in the gel (Fig. 3d). At − 20 and − 70 °C, the protein exhibited 20 and 70% of its activity, respectively (Fig. 3c). Fewer high molecular weight bands in the gel were observed under these storage temperatures, compared to those observed at 4 °C (Fig. 3d). A day after purification without glycerol, a band of ~ 50 kDa of an SDS-PAGE was positively identified as *Vc*IIIPK with a coverage > 70% with other *Vc*PKs by a mass spectrometry MALDI TOF/TOF Analyzer (data not shown). In contrast to *Vc*IPK and *VcIIPK* that are stable either during purification and storage [5], these results confirmed the auto-proteolysis of *Vc*IIIPK. This phenomenon has not been reported in other pyruvate kinases, therefore we are interested in understanding this behavior.

### Conclusions

The genes of *Vc*PKs are a good example of how three paralog genes cannot be transformed and expressed in the same bacterial expression system. Whereas *vcIpk* and *vcIIpk* may be transformed in the 6 strains of BL21 tested, *vcIIIpk* was toxic for BL21(DE3), BL21(DE3)CodonPlus-RIL and BL21-Gold(DE3)pLysS; mildly toxic for BL21(DE3)pLysS and OrigamiB(DE3)pLysS and nontoxic for BL21-AI. This last strain exhibits the lowest basal transcriptional activity avoiding metabolic burden. It was found, that the expression yield for each gene differed from one strain to another. According to [23], the level of toxicity may vary from protein to protein, depending on their physiochemical characteristics. In this respect, in spite of *Vc*IIPK and *Vc*IIIPK being related phylogenetically and exhibiting an identity of 50% in their aminoacid sequences, their protein preservation is drastically different. Whereas one is preserved during purification and storage the other is completely auto-proteolyzed in less than a week. Now we are interested in elucidating the metabolic role of each PK in *V. cholerae*.

## Limitations

In the study of a toxic gene product, a screening of strains should be probed to obtain the best transformation and expression system.

## Additional files

**Additional file 1: Table S1.** Sequence alignment of three PK from *V. cholerae.* Multiple sequence alignment.

**Additional file 2.** Materials and methods.

**Additional file 3: Table S2.** Strains used in this study. Properties of *E. coli* strains commonly used for recombinant protein expression.

**Additional file 4: Figure S1.** Yield efficiency of transformation for *vcIpk*, *vcIIpk* and *vcIIIpk* constructs in different BL21 and XL10-GOLD strains. Competent cells were transformed with 500 ng of DNA of each construct and the colonies grown on the plate were counted (CFU). The star symbol in Origami B(DE3) pLysS and BL21 (DE3) pLysS indicates that the colonies grew after 72 and 24 hours, respectively. In the latter strain different sizes of the colonies were also observed. The error bars represent the standard deviation of three to six independent experiments.

**Additional file 5: Table S3.** The PKs parameters calculated by the ProtParam tool from the ExPASy Bioinformatics Resource Portal (http://web.expasy.org/protparam/).

## Abbreviations

IPTG: isopropyl 1-thio-ß-d-galactopyranoside
LB: Luria Bertani medium
PKs: pyruvate kinases
*Vcpk*: *Vibrio cholerae* pyruvate kinase genes
*Vc*PK: *Vibrio cholerae* pyruvate kinases
