# Supplementary material for: Exploring the differences between the three pyruvate kinase isozymes from Vibrio cholerae in a heterologous expression system
Source: BMC Res Notes. 2018 Jul 31;11:527. doi: 10.1186/s13104-018-3651-8 (PMC6069732; doi:10.1186/s13104-018-3651-8)
Supplement: Supplementary file 1 — Additional file 1: Table S1. Sequence alignment of three PK from V. cholerae. Multiple sequence alignment. [file 13104_2018_3651_MOESM1_ESM.docx]

**Additional File 1:**

Table S1: Sequence alignment^a^ of three PK from *V. cholerae*

| Protein | Identical residues | Similar residues | Percent identity | Percent similarity |
| --- | --- | --- | --- | --- |
| *Vc*IPK_(1-470)_ vs *Vc*IIPK_(1-481)_ | 180 | 93 | 36.81 | 55.83 |
| *Vc*IPK_(1-470)_ vs *Vc*IIIPK_(1-486)_ | 178 | 98 | 35.96 | 55.76 |
| *Vc*IIPK_(1-481)_ vs *Vc*IIIPK_(1-486)_ | 246 | 78 | 50 | 65.85 |

^a^ MAFFT multiple sequence alignment program.
