# Supplementary material for: Exploring the differences between the three pyruvate kinase isozymes from Vibrio cholerae in a heterologous expression system
Source: BMC Res Notes. 2018 Jul 31;11:527. doi: 10.1186/s13104-018-3651-8 (PMC6069732; doi:10.1186/s13104-018-3651-8)
Supplement: Supplementary file 2 — Additional file 2. Materials and methods. [file 13104_2018_3651_MOESM2_ESM.docx]

**Additional File 2- Materials and Methods**

**Plasmid construction and sequencing**

The genome of *V. cholerae* contains three genes encoding for PK, genes *pyk-F* and *pyk-A-1* are in chromosome 1, whereas *pyk-A-2* is in chromosome 2 [1]. The products of these genes are referred as *Vc*IPK*, Vc*IIPK *and Vc*IIIPK*,* respectively [2]. The DNA sequence coding for *pyk-A-2* was obtained from the NCBI database (NC_002506.1) and the gene located in chromosome 2 was obtained using genomic DNA from *V. cholerae* CVD103 strain. This strain was grown overnight at 37 °C in Luria-Bertani (LB), liquid medium without antibiotics, and genomic DNA was purified with the DNAzol Reagent (Invitrogen). The gene (named *vcIIIpk*) was amplified by PCR using the following primers: FW 5´-CATATGTGTAAACCAAAATTGTGG-3´ and RV 5´-GGATCCTTAGGCGAACAGGGC-3´ which contained the restriction sites NdeI and BamHI, respectively. Phusion High-Fidelity DNA polymerase (Thermo FisherScientific) was used and the thermal cycling program was as follow: 25 cycles at 98°C/30 s, 63°C/45 s and 72°C/1.30 min, and then 10 min at 72 °C for the final extension reaction. The amplified fragment (1461 bp) was analyzed using gel electrophoresis in 1% agarose and purified with the Promega Wizard® SV Gel and PCR Clean-Up System (Madison, WI, USA). It was then cloned into the pJET 1.2 vector (CloneJET PCR Cloning Kit; Thermo FisherScientific, Hudson, NH, USA) and transformed into competent *E. coli* XL-Gold cells. To confirm the fidelity of the sequence of the *vcIIIpk* gene, DNA of the plasmid was isolated and completely sequenced. The pJET 1.2 vector containing the verified sequence was digested with the restriction enzymes NdeI and BamHI*, and* subcloned into the pET-HisTEVP expression vector [3]. Finally, the ligation (construct pET-HisTEVP/*vcIIIpk*) was transformed into competent *E. coli* XL10-Gold cells (Stratagene). Plasmid DNA was isolated using the Promega Wizard® SV Minipreps DNA Purification System (Madison, WI, USA) and transformed into different competent *E. coli* BL21 strains (Invitrogen, USA)

**Efficiency of transformation of the *vcpk*s constructs in different *E coli* BL21 strains**

Constructs *pMCSG7/vcIpk*, *pMCSG7/vcIIpk* [2] and pET-HisTEVP/*vcIIIpk* were sequenced with forward and reverse pET-primers in order to confirm the fidelity of each gene. Competent *E. coli* BL21 strains were obtained following the calcium chloride competent cell method [4]. These competent cells were transformed with constructs that were controls for high transformation efficiency (*pMCSG7/vcIpk* and *pMCSG7/vcIIpk*) and with pET-HisTEVP/*vcIIIpk* construct*.* XL10-Gold strain (Stratagene) was also used as a transformation control. Both plasmids *pMCSG7* [5] *and* pET-HisTEVP [3] are based on a pET vector (Novagen, Madison, WI, USA). All the constructs were used to transform six different *E. coli* BL21 strains (Invitrogen, USA) for protein expression: BL21(DE3), BL21(DE3)pLysS, BL21(DE3)CodonPlus-RIL, BL21-Gold(DE3)pLysS, OrigamiB(DE3)pLysS and BL21-AI. Transformation was carried out with 500 ng of each DNA. LB-agar plates, containing the appropriate antibiotic, were grown at 37ºC overnight or the lapse of time required to obtain colonies as described by Sambrook [4]. The total number of colonies was determined (CFU) and the efficiency of transformation was defined as the number of colonies per μg of DNA used. Colonies with *vcIIIpk* construct were grown in cells from the BL21(DE3)pLysS, OrigamiB(DE3)pLysS and BL21-AI strains; whereas no colonies were observed in cells from BL21(DE3), BL21(DE3)CodonPlus-RIL and BL21-Gold(DE3)pLysS strains. To identify if the absence of colonies was related to the latter strains, 200 ng or 500 ng of DNA of *vcIpk* and *vcIIpk* were transformed into the cells of the BL21(DE3), BL21(DE3)CodonPlus-RIL and BL21-Gold(DE3)pLysS strains as controls. To assess whether positive transformants of the *vcIIIpk* construct in these cell strains rely on the amount of DNA, different concentrations were assayed (100, 250, 500 and 1000 ng of DNA). Each transformation reaction was repeated at least three times.

**Growth curves of transformants of *vcpk*s**

Isolated individual colonies containing *vcIpk*, *vcIIpk* or *vcIIIpk* were grown 15 hours in 50 mL of LB medium with the appropriate antibiotics (see Additional File 3: Table S2) at 37 °C and 180 rpm (overnight culture). The next day, fresh LB media with the appropriate antibiotics, were inoculated with the overnight culture of *vcIpk*, *vcIIpk*, and *vcIIIpk* constructs, until OD_600_ reached 0.1. Cultures were grown at 37 °C until OD_600_ reached 0.6. BL21(DE3)pLysS and Origami B(DE3)pLysS cultures were induced with 0.6 isopropyl 1-thio-ß-D-galactopyranoside (IPTG) and BL21-AI cultures with 1.2 mM IPTG plus 0.25% L-arabinose. All cultures were grown in triplicate. Samples were withdrawn from each culture during the bacterial growth every 30 min to measure the absorbance at OD_600_ and build growth curves. In order to ascertain the plasmid stability, 10 mL of the pre-culture, of the culture at 0.6 OD_600_ before induction, and the of culture after 15 h of the addition of the inductors, were taken, and the DNA of *vcIpk* and *vcIIpk* was purified and analyzed digesting it with NdeI and BamHI. The same assay was followed for strains containing *vcIIIpk* except that culture samples of 3 and 6 h after induction were also included. The remaining cells were harvested by centrifugation and stored at -70ºC. Before the cells were harvested, aliquots of 150 μl of the cultures at 0.6 OD_600_ before induction, and 3, 6, 9 and 15 h after induction, were centrifuged and their pellets resuspended in 10 μl of loading buffer and analyzed with SDS-PAGE (12%). Proteins were stained with Coomassie Blue R-250.

**Expression and purification of *Vc*IIIPK**

Four liters of LB BL21-AI strain cells with *vcIIIpk* were induced with 1.2 mM (IPTG) and 0.25% L-arabinose at 25º C for 15 h. The cells were centrifuged and the protein was purified either in the presence or absence of 10% glycerol during the overall purification. Cells were resuspended in lysis buffer (50 mM KH_2_PO_4_ pH 8, 10 mM Imidazole, 300 mM KCl). A complete protease inhibitor EDTA cocktail tablet (Roche Appied Science) and 0.2 mM PMSF were added. Cells were disrupted by sonication using a Sonifier 450 (Branson) for 2.5 min at 40 kHz. The suspension was centrifuged at 13,250 x g, the supernatants loaded on a His Trap FF column (GE Healthcare), and the enzyme eluted with a linear gradient of imidazole (10-500 mM). The fractions with the highest activity of PK were pooled, concentrated using Amicon YM-30 tubes (Millipore Corp., Bedford, MA, USA) and loaded on a Hi Trap desalting column (GE Healthcare) previously equilibrated with 50 mM HEPES, pH 7.5 with or without 10% of glycerol. The purity of the recombinant enzyme was confirmed using SDS-PAGE (12%) stained with Coomassie Blue R-250. Protein concentration was calculated from their absorbance at 280 nm, according to Pace [6], using a molecular absorption coefficient (ε) of 17960 M^-1^cm^-1^. The recombinant enzyme that was purified in the presence of 10% glycerol was stored in 50 mM HEPES with 20% of glycerol at -70ºC, whereas the enzyme purified without glycerol was precipitated with 80% ammonium sulfate and stored at 4 ºC.

Mass spectrometric characterization of *Vc*IIIPK using AB (MDS Sciex) 4800 MALDI TOF/TOF Analyzer (Applied Biosystems) was performed at the Instituto Nacional de Medicina Genómica, México (data not shown).

**Assays of pyruvate kinase activity**

Ammonium sulfate suspensions of lactate dehydrogenase were obtained from Roche Applied Science. Desalted enzymes were prepared as described in Kasahara [7]. The formation of pyruvate was measured spectrophotometrically at 25º C by monitoring the reduction of NADH at 340 nm in a coupled system with lactate dehydrogenase [8]. Reaction mixtures contained 25 mM HEPES, pH 7.0, 3 mM MgCl_2_, 3mM ADP, 5 mM phosphoenolpyruvate and 0.2 mM NADH. One unit of PK activity is the amount of the enzyme required to produce 1 µmol of NAD^+^ per minute under assay conditions, while the specific activity was defined as units per mg of protein. The reaction was initiated with the addition of 100 ng of *Vc*IIIPK recombinant enzyme.

**Protein stability of *Vc*IIIPK during purification and storage**

The specific activity of *Vc*IIIPK and the band pattern in SDS-PAGE (12%) were used to determine the stability of the recombinant enzyme purified and stored in 50 mM HEPES, pH 7.5 with or without glycerol. To test the stability of the enzyme during storage, three conditions were assayed. Aliquots of the enzyme with 20% glycerol were either stored at 4, -20 or -70º C and their activities were determined at 0, 1, 2, 3, 7 and 9 days after storage. On the other hand, 10 µg of protein were loaded onto SDS-PAGE gels after 1, 3 and 7 days of storage. In order to compare the stability of the purified enzyme without glycerol with those purified and stored at three different temperatures with glycerol, an aliquot of the enzyme was stored in 50 mM HEPES pH 7.5 at 4ºC and activities were determined and gels analyzed in the same conditions.

**References for Additional File 2:**

1. Trucksis M, Michalski J, Deng YK, Kaper JB. The Vibrio cholerae genome contains two unique circular chromosomes. Proc Natl Acad Sci USA. 1998; 95: 14464–9.

2. Guerrero-Mendiola C, García-Trejo JJ, Encalada R, Saavedra E, Ramírez-Silva L. The contribution of two isozymes to the pyruvate kinase activity of Vibrio cholerae: One K+-dependent constitutively active and another K+-independent with essential allosteric activation. PLoS One 2017; doi:10.1371/journal.pone.0178673 .

3. Enríquez-Flores S, Rodríguez-Romero A, Hernández-Alcántara G, Oria-Hernández J,. Determining the molecular mechanism of inactivation by chemical modification of triosephosphate isomerase from the human parasite Giardia lamblia: a study for antiparasitic drug design. Proteins. 2011; doi:10.1002/prot.23100.

4. Sambrook JF, Maniatis E. Molecular cloning: a laboratory manual. 2nd ed. New York: Cold Spring Laboratory Press; 1989.

5. Stols L, Gu M, Dieckman L, Raffen R, Collart FR, Donnelly MI. A new vector for high-throughput, ligation-independent cloning encoding a tobacco etch virus protease cleavage site. Protein Expr Purif. 2002; doi: [10.1006/prep.2001.1603](https://doi.org/10.1006/prep.2001.1603).

6. Pace CN, Vajdos F, Fee L, Grimsley G, Gray T. How to measure and predict the molar absorption coefficient of a protein. Prot Sci. 1995; doi: [10.1002/pro.5560041120](https://doi.org/10.1002/pro.5560041120).

7. Kasahara M, Penefsky HS. High affinity binding of monovalent Pi by beef heart mitochondrial adenosine triphosphate. J Biol Chem. 1978; 253:4180-7.

8. Büchner T, Pleiderer G. Pyruvate kinase from muscle. In Colowick S, Kaplan N, editors. Methods Enzymol. New York; Academic Press; 1955. 1:435-440.
