# Supplementary material for: Exploring the differences between the three pyruvate kinase isozymes from Vibrio cholerae in a heterologous expression system
Source: BMC Res Notes. 2018 Jul 31;11:527. doi: 10.1186/s13104-018-3651-8 (PMC6069732; doi:10.1186/s13104-018-3651-8)
Supplement: Supplementary file 3 — Additional file 3: Table S2. Strains used in this study. Properties of E. coli strains commonly used for recombinant protein expression. [file 13104_2018_3651_MOESM3_ESM.docx]

**Additional File 3:**

Table S2: Strains used in this study. Properties of *E. coli* strains commonly used for recombinant protein expression.

| Strains | Relevant characteristics | Key features | Express toxic gene |
| --- | --- | --- | --- |
| XL10-GOLD ^b^ | endA1 glnV44 *recA*1 thi-1 gyrA96 relA1 lac Hte ∆(*mcrA*)183 ∆(*mrr-hsdRMS-mcrBC*)173 tet^r^ F´[proAB *lacI*^q^*Z*∆M15 Tn10(Tet^r^ Amy Cam^r^)] | High transformation with large plasmid inserts |  |
| BL21(DE3)^a^ | F^-^*ompT* *hsd*S_B_(r_B_^-^ m_B_^-^) *gal* dcm (DE3) | Expresses higher levels of heterologous genes | No |
| BL21CodonPlus (DE3)-RIL ^a^ | *endA gal ompT hsdS*_B_ Dcm^+^ Hte Tet^r^ (pACYC-RIL *argU ileY leuW* Cam^r^) | Expresses rare tRNAs;useful for ATrich genomes. Leaky expression of T7 polymerase can lead to uninduced expression of potentially toxic proteins. | Yes |
| OrigamiB(DE3) pLysS ^a^ | F^-^ *ompT* *hsd*S_B_(r_B_^-^ m_B_^-^) *gal dcmlacY1 ahpC (DE3) gor522*::*Tn10* trxB pLysS (Cam^r^, Kan^r^, Tet^r^) | *TrxB gor mutant*, greatly enhanced cytoplasmic disulfide bond formation | No |
| BL21(DE3)pLys^a^ | F^-^ *ompT* *hsd*S_B_(r_B_^-^ m_B_^-^) *gal* dcm(DE3) [pLysS Cam^r^] | Produced T7 lyzosyme to reduce basal level expression of the gene of interest | Yes |
| BL21-Gold (DE3)pLysS ^a,b^ | F^-^ *ompT* *hsd*S(r_B_^-^ m_B_^-^) *dcm*^+^ Tet^+^ gal λ(DE3)endA Hte [pLysS Cam^r^] | Increases transformation efficiency and produces high-quality miniprep DNA | No |
| BL21-AI | F^-^ *ompT hsd*S(r_B_^-^ m_B_^-^) *gal dcm* *araB::T7RNAP-tetA* | T7 RNAP gene under the control of the pBAD promoter. L-arabinose induction. | Yes |

Abbreviations: Kan, kanamycin; Cam, chloramphenicol; Tet, tetracycline. ^a^DE3, lysogen that carries the gene for T7RNA polymerase under control of the *lac*UV5 promoter. ^b^Hte phenotype increases the transformation efficiency. BL21 system is *E. coli* B strains and is deficient in Lon protease (cytoplasm) and OmpT protease (outer membrane) which can degradate proteins during purification.
