# Supplementary figures and images for: Exploring the differences between the three pyruvate kinase isozymes from Vibrio cholerae in a heterologous expression system
Source: BMC Res Notes. 2018 Jul 31;11:527. doi: 10.1186/s13104-018-3651-8 (PMC6069732; doi:10.1186/s13104-018-3651-8)

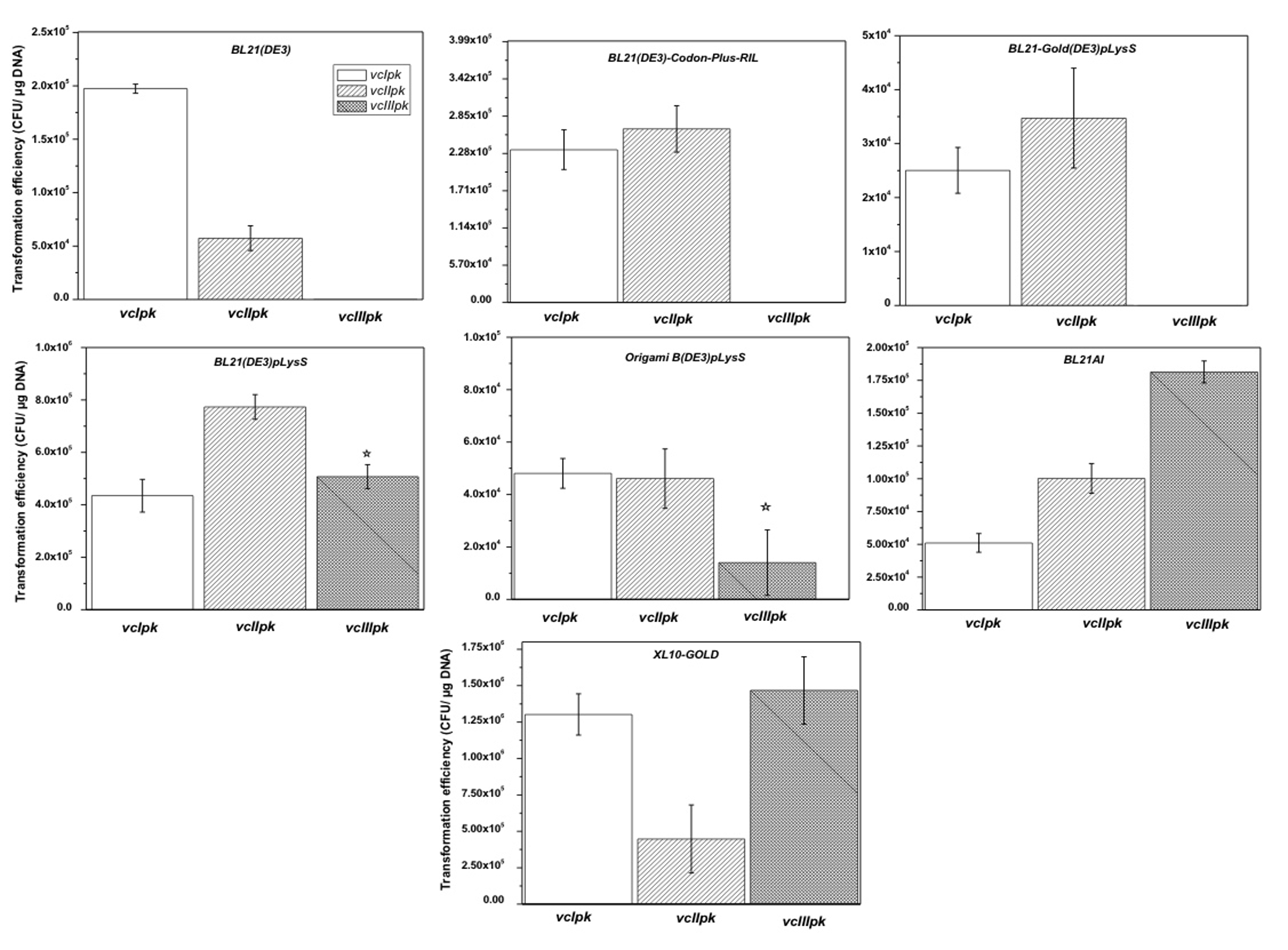

Supplement: Supplementary file 4 — Additional file 4: Figure S1. Yield efficiency of transformation for vcIpk, vcIIpk and vcIIIpk constructs in different BL21 and XL10-GOLD strains. Competent cells were transformed with 500 ng of DNA of each construct and the colonies grown on the plate were counted (CFU). The star symbol in Origami B(DE3) pLysS and BL21 (DE3) pLysS indicates that the colonies grew after 72 and 24 hours, respectively. In the latter strain different sizes of the colonies were also observed. The error bars represent the standard deviation of three to six independent experiments. [file 13104_2018_3651_MOESM4_ESM.jpg]
