# Supplementary material for: Exploring the differences between the three pyruvate kinase isozymes from Vibrio cholerae in a heterologous expression system
Source: BMC Res Notes. 2018 Jul 31;11:527. doi: 10.1186/s13104-018-3651-8 (PMC6069732; doi:10.1186/s13104-018-3651-8)
Supplement: Supplementary file 5 — Additional file 5: Table S3. The PKs parameters calculated by the ProtParam tool from the ExPASy Bioinformatics Resource Portal (http://web.expasy.org/protparam/). [file 13104_2018_3651_MOESM5_ESM.docx]

**Additional File 5:**

Table S3: The PKs parameters calculated by the ProtParam tool from the ExPASy Bioinformatics Resource Portal (<http://web.expasy.org/protparam/)>.

| Protein | Number of amino acids | Mw (Da) | pI | Trp residues | Cys residues |
| --- | --- | --- | --- | --- | --- |
| VcIPK | 470 | 50,439.14 | 5.79 | 0 | 5 |
| VcIIPK | 481 | 52,333.29 | 5.66 | 0 | 5 |
| VcIIIPK | 486 | 52,111.9 | 5.44 | 2 | 8 |
